# Supplementary material for: Computer-Aided Diagnosis Improves the Detection of Clinically Significant Prostate Cancer on Multiparametric-MRI: A Multi-Observer Performance Study Involving Inexperienced Readers
Source: Diagnostics (Basel). 2021 May 28;11(6):973. doi: 10.3390/diagnostics11060973 (PMC8227686; doi:10.3390/diagnostics11060973)
Supplement: Supplementary file 1 [file diagnostics-11-00973-s001.zip › diagnostics-1222240-supplementary.pdf]

Supplementary Tables

Supplementary Table S1: MRI sequence details for the different types of acquisitions

Sequence	ST (mm)	FOV (cm)	NEX	AM	RM	TR/TE/FA	Additional information
T2w (fast spin echo - FSE)	3	16x16	2	384x288	512x512	3020/85/160°	
DW (echo planar imaging - EPI)	3	16x16	14	128x128	256x256	7000/101/90°	b-values: 0-800 s/mm^2
DW (single shot echo planar imaging - FOCUS)	3	16x16	16	140x70	256x256	3200/73/90°	b-values: 0-1000 s/mm^2; %FOV = 50
DCE (3D Spoiled Gradient echo –SPGR)	3	20x20	0.5	224x192	512x512	3.6/1.3/20°	temporal resolution = 13 s; 26 timepoints

ST = slice thickness; FOV = field of view; NEX = number of executions; NEX = number of excitations; AM = acquisition matrix; RM = reconstruction matrix; TR/TE/FA = repetition time/echo time/flip angle

Supplementary Table S2: Per patient negative and positive predictive values for unassisted and assisted readings. 95% confidence intervals are reported in squared brackets.

	**Unassisted reading**	**Assisted reading**	***p* value**
**Negative predictive value**			
Reader 1	72.9 (43/59) [64.3-80.0]	73.8 (45/61) [65.5-80.6]	0.456
Reader 2	71.0 (44/62) [63.0-77.8]	69.2 (36/52) [59.7-77.4]	0.417
Reader 3	80.4 (41/51) [70.2-87.7]	83.3 (40/48) [72.6-90.4]	0.355
Average	74.4 (128/172) [69.5-78.8]	75.2 (121/161) [69.9-79.8]	0.433
**Positive predictive value**			
Reader 1	93.5 (29/31) [78.6-98.3]	100.0 (29/29)	0.083
Reader 2	96.4 (27/28) [79.3-99.5]	76.3 (29/38) [63.3-85.7]	**0.013**
Reader 3	89.4 (35/39) [77.2-95.8]	88.1 (37/42) [76.2-94.5]	0.427
Average	92.9 (91/98) [86.2-96.4]	87.2 (95/109) [80.3-91.9]	0.087
